# A method for the controllable fabrication of optical fiber-based localized surface plasmon resonance sensors

**DOI:** 10.1038/s41598-022-13707-y

**Published:** 2022-06-10

**Authors:** Alba Calatayud-Sanchez, Angel Ortega-Gomez, Javier Barroso, Joseba Zubia, Fernando Benito-Lopez, Joel Villatoro, Lourdes Basabe-Desmonts

**Affiliations:** 1 Microfluidics Cluster UPV/EHU, BIOMICs Microfluidics Group, Lascaray Research Center, University of the Basque Country UPV/EHU, Vitoria-Gasteiz, Spain; 2 Department of Communications Engineering, University of the Basque Country UPV/EHU, Bilbao, Spain; 3 Microfluidics Cluster UPV/EHU, Analytical Microsystems and Materials for Lab-On-a-Chip (AMMa-LOAC) Group Analytical Chemistry Department, University of the Basque Country UPV/EHU, Leioa, Spain; 4 BIOARABA Health Research Institute, Microfluidics Cluster UPV/EHU, Vitoria-Gasteiz, Spain; 5 BCMaterials, Basque Center for Materials, Applications and Nanostructures, UPV/EHU Science Park, Leioa, Spain; 6 IKERBASQUE, Basque Foundation for Science, Bilbao, Spain

**Keywords:** Optics and photonics, Optical materials and structures, Optical techniques, Other photonics

## Abstract

Optical fiber-based Localized Surface Plasmon Resonance (OF-LSPR) biosensors have emerged as an ultra-sensitive miniaturized tool for a great variety of applications. Their fabrication by the chemical immobilization of gold nanoparticles (AuNPs) on the optic fiber end face is a simple and versatile method. However, it can render poor reproducibility given the number of parameters that influence the binding of the AuNPs. In order to develop a method to obtain OF-LSPR sensors with high reproducibility, we studied the effect that factors such as temperature, AuNPs concentration, fiber core size and time of immersion had on the number and aggregation of AuNPs on the surface of the fibers and their resonance signal. Our method consisted in controlling the deposition of a determined AuNPs density on the tip of the fiber by measuring its LSPR signal (or plasmonic signal, Sp) in real-time. Sensors created thus were used to measure changes in the refractive index of their surroundings and the results showed that, as the number of AuNPs on the probes increased, the changes in the Sp maximum values were ever lower but the wavelength shifts were higher. These results highlighted the relevance of controlling the relationship between the sensor composition and its performance.

## Introduction

Localized Surface Plasmon Resonance (LSPR) is the resonance of free electrons on a noble metal (usually gold or silver) nanostructure when excited by a light whose wavelength is larger than their size. This coherent oscillation results in a unique scattering and absorption spectra that depends on the composition, size, shape and surface density of the nanostructures but also on the chemical and physical characteristics of their surroundings, rendering a signal whose maximum reached a specific value and was positioned at a specific wavelength^1^. LSPR-based sensors take advantage of this sensitivity to measure target-induced modifications in the immediate environment of the metal nanostructures such as refractive index changes, plasmonic-molecular coupling or nanoparticle growth; although the former is the most extensively used^2^, measured as either wavelength shifts or changes in the signal maximum values. Due to their label-free, ultrasensitive nature, this kind of sensors have been of increasing interest in the biosensing field^3–6^.

Optical fibers (OFs) transmit light signals between two points thanks to their total internal reflection due to the difference between the refractive indexes of their core and cladding. As sensors, OFs provide a myriad of advantages, including their miniature size and flexibility, which make them greatly versatile tools to implement in portable devices along with a simple optical set-up^7^. These properties, in addition to their high electromagnetic immunity, durability, remote sensing capabilities, cost-effectiveness and reliability, have placed them as excellent platforms for chemical- and bio- sensing^8–11^. When OFs are combined with the LSPR effect, ultrasensitive and ultra-small OF-LSPR biosensors can be obtained^12–18^.

There are three main groups of configurations in which all the different types of optical fibers can be exploited as biosensors: through direct excitation of their end face^19^, through the exposition of their core on their longitudinal dimension^20^ or through the resonant coupling of internal grattings^21,22^. In this study, the former strategy is chosen, which provides an enhanced light-sample interaction, is less complex to fabricate and allows sensor regeneration by simply cleaving the optical fiber^23,24^.

Although lithographic methods are consolidated as the main option to nanostructure the facet of an optical fiber and to fabricate other type of nanospectroscopic platforms, they can be time-consuming, complex and expensive, due to the high cost and the bulky size of the equipment required^25–31^. In contrast, chemical immobilization of gold nanoparticles (AuNPs) on the end face of an optical fiber results on a much simpler and cost-effective process. Kajikawa and co-workers described this method for the first time^32^, and it has been widely used in the last decade for many biosensing applications^33–40^. This method consists in the attachment of colloidal AuNPs to a self assembled monolayer (SAM) by immersion of the functionalized fiber facet in a AuNPs suspension, which requires less equipment and is more versatile (it can be used with any type of NPs).

However, this chemical immobilization on the fiber facet surface is difficult to control since AuNPs can reach several degrees of aggregation (monomers, dimers, trimers, etc.), directly affecting the shape of the LSPR signal, as well as the position and intensity of its maximum value, and hence, the performance of the OF-LSPR sensor. The intensity of the LSPR signal increases with the size and number of AuNPs on the optical fiber face (which could be described as a higher surface density ratio), however, this parameter does not directly correlate with the sensitivity of the sensor.

Jeong et al. described how the size and surface density ratio of AuNPs on the OFs facets are inversely proportional to the sensor sensitivity, it is to say, the smaller the AuNPs and the lower the coverage of the OF surface, the higher the sensitivity^41^. They controlled the density of AuNPs on the fiber during the fabrication process by varying the time of immersion in the AuNPs solution and observed that fibers with a higher AuNPs density presented lower sensitivity to changes in the refractive index. This sensitivity was measured through the changes in the maximum values of the LSPR signal. The loss in sensitivity was correlated with the amount of aggregated AuNPs on the tip of the fiber, which increased with the time of immersion in the AuNPs solution. This way, it was found that a surface density ratio of 45% rendered the optimal results, because at lower coverages the signal was unstable^41^.

Nevertheless, in the work of Jeong et al*.* it was also highlighted that the randomness in the immobilization process made it difficult to properly control the amount of immobilized AuNPs, even in batch-prepared probes^42^, which reduced the linearity and overall reliability of the measurements, a crucial feature in the development of biosensors. A signal calibration system was proposed where the LSPR signal obtained after the fabrication of the sensor was used as the baseline value^38^. Taking into account that the behavior of the OF-LSPR sensor changes with its composition, grouping and analyzing probes with varying surface characteristics could render misleading results.

**Figure 1:**
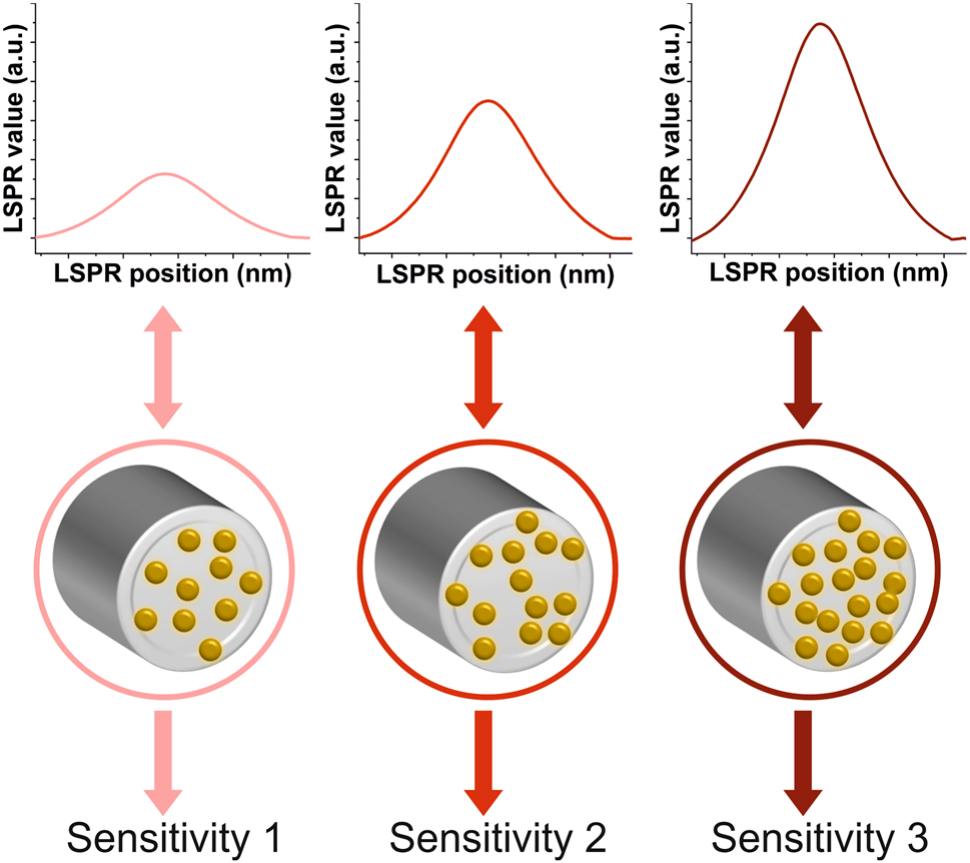
Scheme of the influence of the OF-LSPR sensor composition on its LSPR signal and performance. The density and degree of aggregation of the AuNPs immobilized on the facet of an optical fiber can be deduced from its LSPR signal. OF-LSPR sensors with different LSPR peak values render different sensitivities upon changes in their surroundings.

Therefore, there is a need for a method to prepare ultrasensitive nanosensors based on AuNPs-coated optical fiber (OF-LSPR sensors) with reproducible composition. Herein, we propose to produce OF-LSPR sensors with similar performances by monitoring in real time the LSPR signal as AuNPs are being immobilized on the surface of the optical fiber end face. This way, many probes can be easily fabricated with similar number of AuNPs on their surface (Fig. 1).

For this purpose, we have carried out three main approaches. First, in order to provide further insight into the factors that affect AuNPs immobilization, we have studied the effect of temperature and AuNPs concentration on the temporal evolution of the LSPR (or resonance) signal. Moreover, the effect of using fibers with different core sizes, which helps profiling the fabrication protocol that best suits each assay was also investigated. Secondly, we performed a detailed microscopic analysis of the end facets of fibers with different densities of immobilized AuNPs and related them to the peak value of the LSPR signal. And thirdly, in order to determine how these differences in the final composition of the sensors affected their performance, a final study was carried out to compare the refractive index sensitivity of optical fibers with different values of resonance intensities, not only analyzing the changes in that peak value but also its wavelength shift.

## Results and discussion

### Effect of temperature, AuNPs concentration and fiber core size on resonance signal

In order to characterize the effect of external factors on the chemical immobilization of AuNPs onto the tip of OFs, the temporal evolution of the LSPR signal was measured under various conditions: different temperatures, different AuNPs concentration and different fiber core diameters.

**Figure 2:**
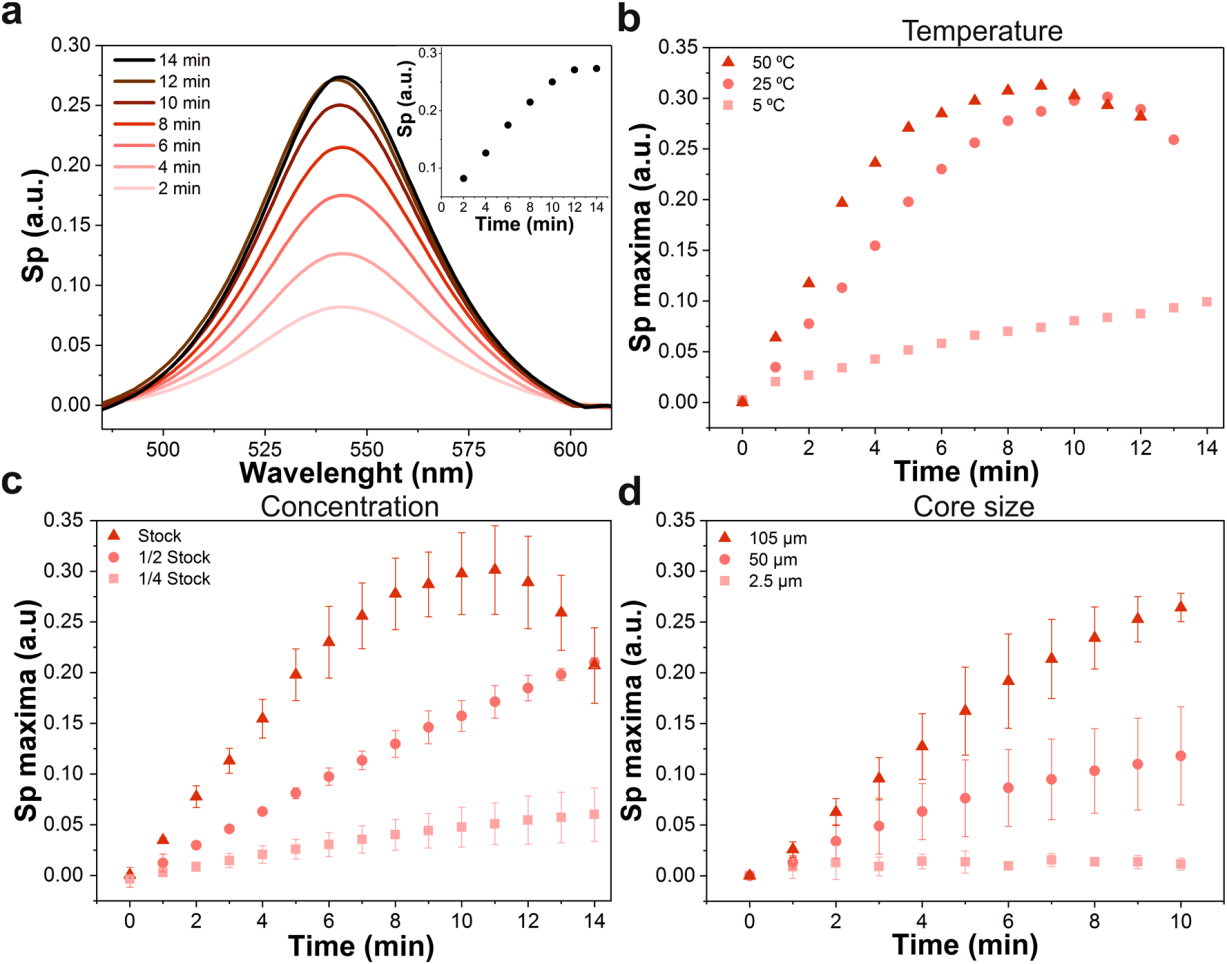
Effect of temperature, AuNPs concentration and optical fiber core size on the LSPR signal of OF-LSPR sensors. (**a**) Temporal evolution of the LSPR signal of one 105MMF immersed in stock concentration of AuNPs at room temperature (22 °C). (**b**) Sp maximum values for 105MMFs at different temperatures (5, 25, and 50 °C) measured every minute during 14 min after immersion in AuNPs. (**c**) Sp maximum values achieved at 22 °C and fiber core diameter of 105 μm, with different AuNPs concentrations, measured every minute during 14 min after immersion in AuNPs. (**d**) Sp maximum values for optical fibers with different core diameters (2.5, 50 and 105 μm), where AuNPs were chemically immobilized up to 10 min. Error bars correspond to the standard deviation of the measurements in three different optical fibers (n = 3).

Optical fibers were functionalized with a SAM of (3-Aminopropyl)triethoxysilane (APTES) so that their glass surface presented amino groups that interacted with the colloidal AuNPs (40 nm in diameter) in suspension while the plasmonic signal was monitored in real time (Fig. 2a). In order to obtain this signal, white light from a LED was used to excite the AuNPs that were being immobilized on the tip of the OF. The light that travelled back to the spectrometer was processed and expressed as Sp, the result of a logarithmic expression that comprises all the changes in light due to the presence of the AuNPs on the fiber (Eq. 1).

1 $$Sp\;{ }(a.u.) = { } - log\left( {\frac{S - D}{{R - D}}} \right)$$

In Eq. (1), S is the spectrum recorded when AuNPs are immobilized, R is the obtained spectrum when there are no AuNPs onto the optical fiber facet and D is the spectrum recorded when the LED is switched off. Sp values were expressed in arbitrary units (a.u.).

Regarding the effect of the different parameters, firstly, three different fibers (three multimode fibers with a 105 µm core diameter, 105MMF) were immersed in AuNPs (at stock concentration) while submitted to three different environmental temperatures: 5, 25, and 50 °C (Fig. 2b). Parallel to these experiments, another set of fibers were exposed to different concentrations of AuNPs: the stock one (3 × 10^10^ particles·mL^−1^), 1/2 the stock concentration, and 1/4 of the stock concentration; at constant room temperature (22 °C) (Fig. 2c). A third group of experiments consisted in using MMFs of different core sizes—105 and 50 µm—and single mode fibers with a core of 2.5 µm to be immersed in the stock concentration of AuNPs at constant room temperature (Fig. 2d). The impact of these three variables was evaluated on the AuNPs immobilization and their LSPR spectra through the maximum values of Sp, measured each minute during 10–14 min.

Figure 2a shows an example of the general temporal evolution of the resonance signal observed in every case, which follows a distinct tendency. At shorter times, the maximum value of Sp increased linearly; when the fiber was immersed for longer times, this value reached a plateau, which was maintained for a couple of minutes, and then it started diminishing. A red shift of the LSPR spectrum was also observed (Fig. 2a). However, the time it took for the maximum Sp value to get to the plateau depended on the combination of the tested parameters.

The chemical factors—temperature and AuNPs concentration—showed the expected effect of accelerating the reaction. When the temperature rises, a faster kinetic is expected, by increasing the interactions between the AuNPs and the end face of the optical fibers resulting in a faster immobilization, hence reaching the saturation point faster at higher temperatures. More AuNPs in the same volume (higher concentrations) had the same result. Regarding the probe fabrication with fibers of different core sizes, the smaller the core diameter, the faster the signal saturation was reached and the lower the achieved maximum Sp. This does not mean that the speed of immobilization varied, but rather that, as the fiber core was smaller, so was the area to be covered by AuNPs; thus, the surface got saturated earlier and the number of AuNPs that contributed to the total LSPR resonance was lower. Therefore, wider fiber cores render a broader dynamic range to fabricate OF-LSPR probes to be used for sensing. Moreover, fibers with a bigger core size provide a higher binding capacity, which can improve the sensitivity of the sensor when used for biosensing through the measurement of target-binding induced changes on the Sp maximum value.

These results highlight the importance of a proper design protocol before developing an OF-LSPR sensor. For the following experiments, constant conditions of temperature (22 °C), AuNPs suspension concentration (stock, 3 × 10^10^ particles·mL^−1^) and optical fiber core size (105 µm MMF) were employed.

### SEM characterization: correlation between AuNPs density and LSPR signal

Once the effect of the external conditions on the LSPR spectrum was defined, the AuNPs immobilization on the optical fiber was analyzed by SEM imaging in order to obtain the relation between the number and aggregation of the nanoparticles and their LSPR signal.

**Figure 3:**
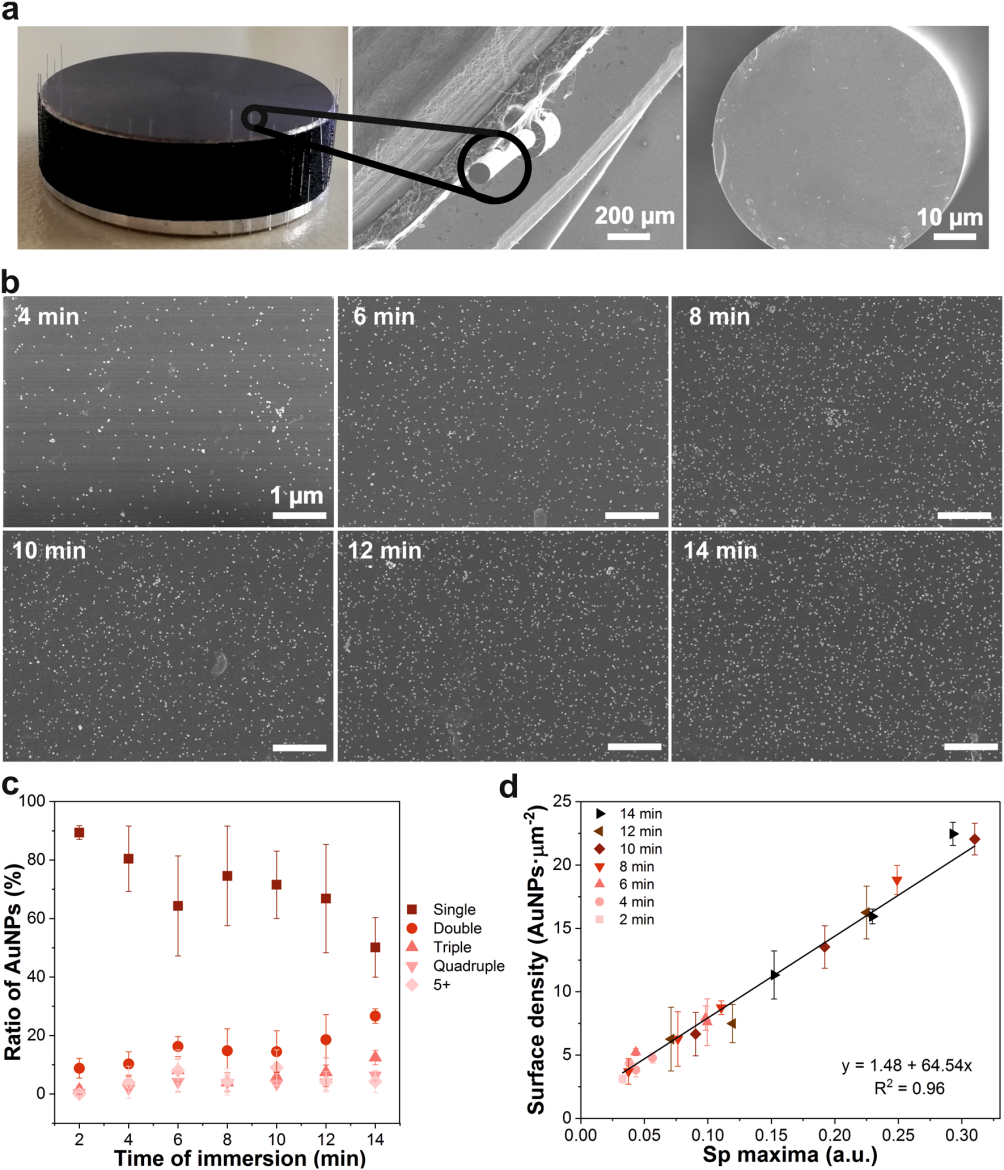
SEM characterization: correlation between AuNPs surface density and LSPR signal (Sp maximum values). (**a**) Pictures of the optical fibers stuck on the SEM sample holder, the view of a single optical fiber inside the SEM chamber and a detail of the fiber’s end face. (**b**) Representative SEM images for every immersion time (scale bar 1 µm). (**c**) AuNPs distribution values regarding whether each AuNPs was alone (single) or in contact with others (double, triple, quadruple and five or more aggregated nanoparticles) plotted against their respective times of immersion; error bars correspond to the standard deviation of the number of AuNPs present on the end faces from three different optical fibers for each immersion time (n = 3, except at 2 min, where only one fiber is represented with the deviation between its three pictures). (**d**) AuNPs surface density (nanoparticles per square micrometer) of the fibers immersed for different times, plotted against their respective Sp maximum values. Error bars correspond to the standard deviation of the number of AuNPs in three different SEM images from each optical fiber (n = 3).

For this purpose, a series of MMFs were functionalized as previously described and immersed in a solution of AuNPs for different times: 2–14 min in intervals of 2 min. After the immobilization, their Sp values were recorded and then they were taken to be imaged by a SEM (Fig. 3a,b). Three 100 µm^2^ SEM images of each of the end faces of the fibers were taken and analyzed to obtain two sets of data. First, the proportions of the total AuNPs that were immobilized with different degrees of aggregation: single, double, triple, quadruple, and five or more joined AuNPs, after each immobilization time (Fig. 3c, Fig. SI1). Second, the total number of immobilized AuNPs per area (or surface density), which was then plotted against their respective maximum Sp values (Fig. 3d).

Regarding the disposition of the AuNPs, it was observed that at shorter times of incubation there was a predominant percentage of single nanoparticles (up to 90% at 2 min), whereas, at longer times, this population of AuNPs decreased considerably (down to 50% after 14 min). However, at intermediate times, the proportions of single and aggregated AuNPs could be considered to stay equivalent. These results correlated with the fact that there is not a linear association between the total number of AuNPs on the end face of the optical fibers and the time they were immersed in the solution, especially at those intermediate time intervals, as it can be appreciated in Fig. 3d.

These observations agree with the fact that the employed AuNPs immobilization methodology is a chemical process governed not only by time, but by all parameters affecting chemical reactions such as temperature and reagent concentration. In addition, this behavior may explain the saturation of the LSPR signal observed in Fig. 2a, as this signal is directly correlated with the number of immobilized AuNPs up to a point when a metallic state is reached and the plasmonic effect steadily turns into an increased reflection of the light.

Actually, a linear relationship was found when plotting the AuNP surface density of each probe against their respective Sp maximum, highlighting the power of this value—which can be measured in real time—as a reference to obtain sensing probes with the same characteristics. Furthermore, from this linear correlation we could calculate the minimum amount of AuNPs that could be detected by our system (2.125 AuNPs µm^−2^), which corresponds to a total of 18,404 AuNPs on the surface of the core of a 105MMF.

### LSPR signal (Sp) values as reference to fabricate plasmonic probes with comparable performances

#### Reproducibility of the probes

**Figure 4:**
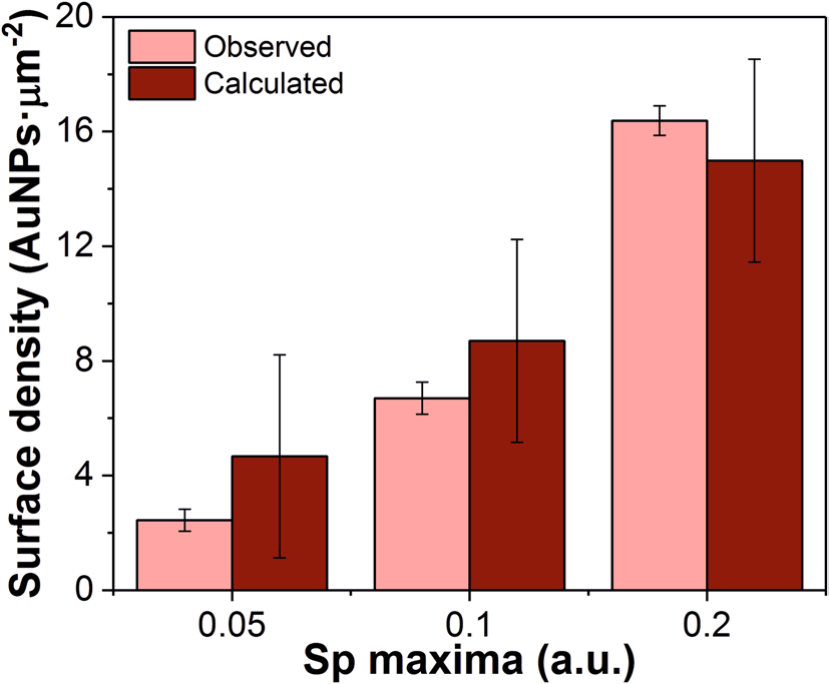
AuNPs density of probes with different initial Sp maximum values. Observed and calculated AuNPs surface density of fibers fabricated with three different Sp maximum values: 0.05, 0.10 or 0.20 a.u. (n = 3).

Once it was confirmed that there is a linear correlation between the Sp maximum value and the AuNP surface density on a OF-LSPR—but not with time, due to the difficulty to control all parameters influencing the chemical process—an experiment was performed to demonstrate that several sensors with the same AuNP density could be easily fabricated by monitoring Sp, independently of other factors. Nine sensors with three different AuNPs densities were fabricated by stopping the immobilization reaction at signals whose Sp maximum values were 0.05, 0.1 and 0.2 a.u. These tips were also imaged by SEM (see Fig. SI2). The number of AuNPs on their surfaces were counted and compared to the expected value estimated from the linear equation on Fig. 3d: y = 1.48 (± 0.32) + 64.54 (± 3.21)x; where y is the amount of AuNPs per square micrometer (AuNPs surface density) and x is the maximum value of Sp. Figure 4 shows the comparison between the calculated and the observed values at the three different Sp maxima.

The accuracy of this method was calculated to be low for all the samples, but it increased with the Sp value, reaching a 9% for 0.2 a.u. This means that the capacity of our method to predict the number of AuNPs immobilized on the end face of the optical fiber from the value of its Sp maximum gets higher as the density of AuNPs increases. Moreover, it was confirmed that a very low variability between samples could be achieved by controlling the Sp maximum during the AuNPs immobilization.

#### Sensitivity of the probes

Finally, in order to evaluate how different compositions of the sensors can affect their performance, their sensitivity to changes in the refractive index (RI) of their surroundings was tested against the number of AuNPs on their surface. A series of OF-LSPR sensors with a Sp maximum in water ranging from 0.01 to 0.30 were fabricated by monitoring their signal in real time during the AuNPs immobilization. Then, they were exposed to solutions of water:glycerol with increasing RI values: from 1.33 to 1.37 measured in RIUs (refractive index units).

**Figure 5:**
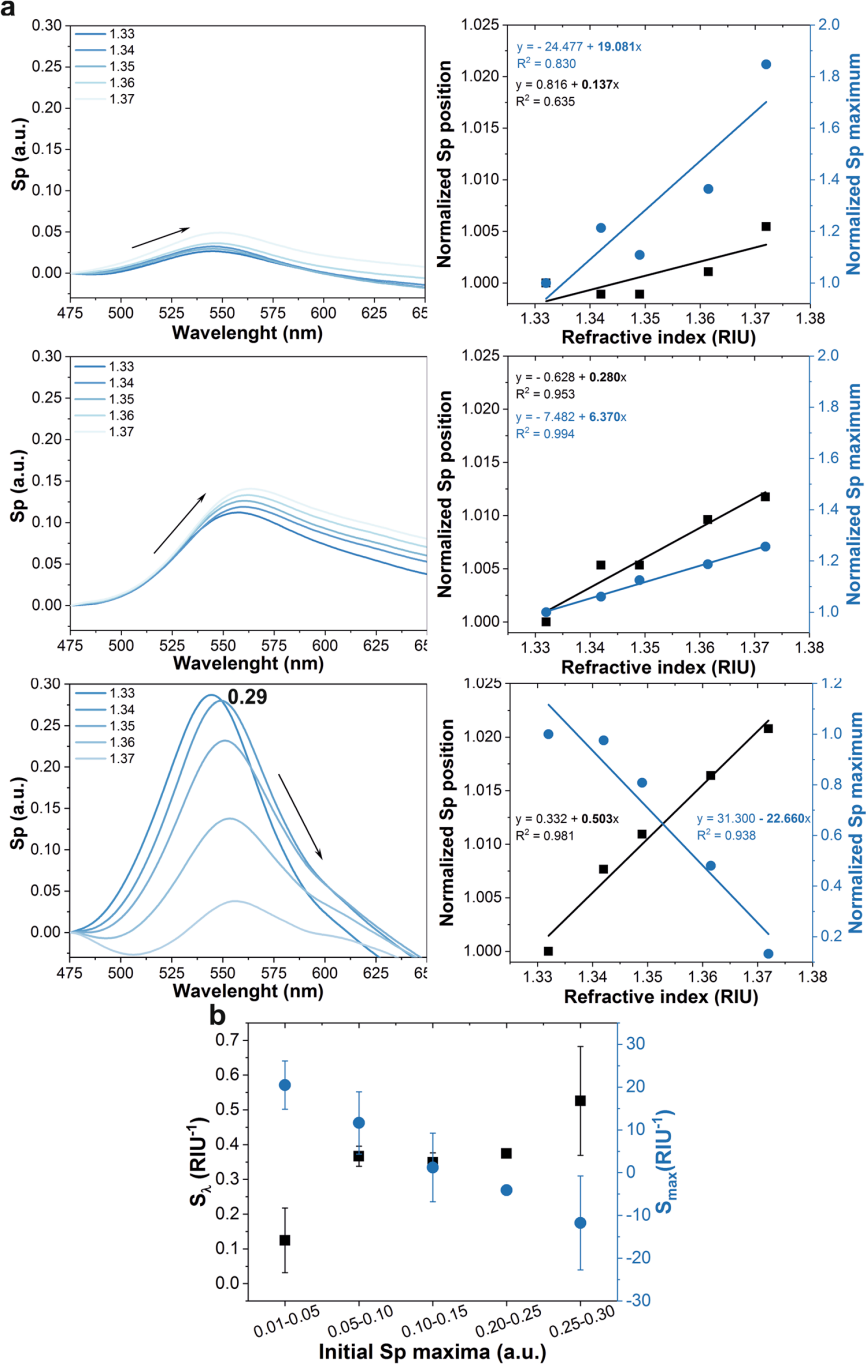
Sensitivity to changes in the refractive index of probes with different initial Sp maxima. (**a**) Examples of the performance of fibers with initial Sp maxima of 0.03, 0.11 and 0.29 a.u. On the right column, the evolution of the normalized wavelength position and value of the Sp maxima upon exposure to increasing RI solutions. (**b**) S_λ_ and S_max_ values obtained from the slopes of the linear regressions of the results from fibers with different initial Sp maxima. The measured fibers were grouped in five sets according to their range of initial Sp maximum values. Error bars correspond to the standard deviation between the fibers in each set (n = 4, except for the range of 0.2 to 0.25 a.u., with only one replica).

The LSPR signal of this kind of sensors is affected by alterations in their immediate environment, so changes in the position and value of the Sp maxima can be measured as the RI of the solution increases or decreases (Fig. 5). For their application on biosensing, RIs from 1.33 to 1.37 are the most interesting, given that 1.33 is the approximate RI of water and biomolecules interacting with the sensor surface can increase that value up to 1.37. This phenomenon is the basis of most LSPR biosensors^3^. In this experiment, both the peak position (wavelength, λ, in nm) and maximum values of Sp (in a.u.) were measured when immersed in each RI solution (n) and then normalized taking those values at a RI of 1.33 as reference (Eqs. 2 and 3).

2 $$Normalized\; Sp \;position = \frac{{\lambda_{n} }}{{\lambda_{1.33} }}$$

3 $$Normalized \;Sp\; maximum = \frac{{Sp_{n} }}{{Sp_{1.33} }}$$

The sensitivity of each OF-LSPR sensor was defined as the change in either the change in position or value of the maximum Sp per unit change of the RI (RIU) and expressed as a function of the normalized values for those parameters (S_λ_ and S_max_, respectively) as shown in Eqs. (4) and (5). These values coincided with the slopes of the linear regressions of the normalized position and maximum Sp against the tested RIs (Fig. 5a, right column, in bold letters).

4 $$S_{\lambda } \;(RIU^{ - 1} ) = \frac{Normalized \;Sp\; position }{{\Delta_{n} RIU}}$$

5 $$S_{max} \;(RIU^{ - 1} ) = \frac{Normalized \;Sp \;maximum}{{\Delta_{n} RIU}}$$

The results indicate that the amount of AuNPs on the sensor’s tip did indeed have an effect on its response to changes in the RI. Figure 5a shows three examples: two probes in the extreme cases with either very few AuNPs on the fiber end face (low AuNPs density) or very high AuNPs density (Sp maxima of 0.03 and 0.29 a.u., respectively) and one in an intermediate state (Sp maximum of 0.11 a.u.).

Interestingly, the changes in sensitivity when measuring either the changes in the position or in the value of the Sp maxima followed opposite tendencies as the surface density of AuNPs on the probes augmented. On the one hand, sensors with a low AuNPs density provided a high sensitivity regarding Smax, but low sensitivity when evaluating Sλ. In contrast, when using sensors with higher amounts of AuNPs, the changes in the maximum values of the Sp decreased (lower Smax), their values reaching even negative values (indicating that the Sp signal was reduced with increasing RIs), while the wavelength shifts increased (higher Sλ). These tendencies can be better recognized in Fig. 5b, which shows the values of S_λ_ and S_max_ obtained from the linear regression of fibers with different initial Sp maxima in five ranges: from 0.01 to 0.05, 0.05 to 0.10, 0.10 to 0.15, 0.15 to 0.20, 0.20 to 0.25 and 0.25 to 0.30 (all in a.u.).

Some LSPR biosensors rely on wavelength shifts and others on the changes in the Sp maxima, depending on the application. The findings of this work manifest how the OF-LSPR end face composition (in terms of AuNPs density and aggregation) can distinctly alter the response of the sensor for both kinds of measurements, providing, to the best of our knowledge, previously unknown information to the users.

## Conclusions

The results of this work provide the users of fiber-optic based LSPR sensors fabricated by the chemical immobilization of AuNPs on the fiber’s end face with three main points of information. Firstly, environmental conditions such as temperature and AuNPs concentration must be controlled in order to reduce the variability of the fabrication process. Additionally, optical fibers with bigger core diameters provide a broader dynamic range than smaller or single mode fibers, thus enabling the fabrication of OF-LSPR sensors with a wider variety of properties. Secondly—and in order to add to that control, which cannot be nanometrically managed, resulting in unwanted aggregations—the surface density of AuNPs on the sensor does not depend solely on the time of immersion of the functionalized fibers on the AuNPs suspension, but it can be estimated by measuring the Sp maximum of the LSPR signal. And thirdly, the density of AuNPs on the surface of the probe affects the sensitivity of the sensor towards changes in the RI of its surroundings, not only in its magnitude but on which parameter it alters more: the position of the Sp maximum or its value. It must be noted that the reported results have only been validated with AuNPs of a diameter of 40 nm and different sizes may present some differences in behaviour. However, this diameter was chosen as the optimal size in order to create OF-LSPR sensors in which the contribution of absorbance effects on the signal are higher than those of scattering whilst also providing the best surface to volume ratio for interacting with the analytes.

In order to ensure the best reproducibility possible (regarding the number of AuNPs on the fiber’s facet and the sensitivity of the sensors), we propose to control the one variable that can be completely monitored in real-time: the LSPR signal, whose wavelength position and peak value can be tracked over time with high accuracy.

This unprecedented finding constitutes a progress in the understanding of the fabrication of OF-LSPR sensors on the facet of the fibers, which will help future users to obtain better results in their findings without the need of exhaustive characterizations prior to their use.

## Materials and methods

### Chemicals

All chemicals, namely: (3-Aminopropyl)triethoxysilane (APTES), sulphuric acid (H_2_SO_4_, ACS reagent, 95.0–98.0%), hydrogen peroxide (H_2_O_2_, 30%), isopropanol (IPA, ACS reagent ≥ 99.5%) and glycerol (C_3_H_8_O_3_, 99%, GC); were purchased from Merck KGaA (Spain Branch Division) and used as received from the supplier. Citrate stabilized 40 nm diameter spherical gold nanoparticles (AuNPs) solution was purchased from Nanovex Biotechnologies S.L. (Spain) at a concentration of 3 × 10^10^ particles·mL^−1^, which was reduced to 1.5 × 10^10^ and 7.5 × 10^9^ particles·mL^−1^ with MilliQ water to study the effect of the nanoparticle concentration on the LSPR spectrum. The AuNPs were stored at 4 °C and kept in the dark. AuNPs of this size (40 nm) were selected because it provides.

### Equipment and settings

The optical set-up used in this work consisted of a LED (MCWHL5, Thorlabs) as the light source in the 400–700 nm range (which matches the resonance wavelength of the AuNPs used), a fiber optical coupler (FOC) and a mini-spectrometer (Avantes, mini2048-VI25) (Fig. SI3). In order to study the effect of the core size in the obtained signal, three different fibers were used with their correspondent couplers. Two multimode fibers (MMF) with a core diameter of 105 µm and 50 µm (FG105LCA and FG050LCA, Thorlabs). Herein, such fibers will be denoted as 105MMF and 50MMF, respectively. The FOCs for the 105MMF and 50MMF were, respectively, a TM105R5S1A and TM50R5S2A purchased from Thorlabs. The other fiber used was a single mode fiber (SMF) in the visible range with a core diameter of 2.5 µm (460HP, Thorlabs) with a FOC (TW560R5F2, Thorlabs).

All the optical fibers were cleaned and cleaved using an optical fiber cleaver (VF-78, INNO Instrument America).

### AuNPs immobilization

In order to attach the AuNPs to the optical fibers end face, a previously described methodology^38^ was applied in each of the experiments described in this paper. Briefly, the optical fibers were treated with a piranha solution (H_2_SO_4_:H_2_O_2_, 3:1) for 30 min, to clean and oxidize the glass so that the surface is activated for the reaction in the next step. Piranha solution releases irritating vapors and is very corrosive to most materials, therefore it must be carefully handled and stored, working in a fume hood and placing it in resistant containers such as glass vials. After rinsing with water:ethanol (1:1) and let dry, the optical fibers were immersed in a 5% APTES IPA solution for 90 min, in the dark. Later, the optical fibers were rinsed in an IPA-water (1:1) solution and let dry. Finally, the optical fibers were immersed in the AuNPs solution to immobilize them on the tip. The immobilization process is summarized in Fig. SI4, where the impact of each step on the optical spectrum is shown as a reference.

### LSPR spectra collection

The light is launched from the LED through the FOC to the end face of the optical fiber, where the AuNPs are located. Then, the light is reflected by the optical fiber end face, which acts as a low reflectivity mirror and it is where the AuNPs are immobilized. The interaction of the light with such nanoparticles triggers the LSPR effect. The reflected light passes again through the FOC, finally arriving to the spectrometer, which displays the received signal as Sp, whose value is obtained by applying the following expression:

1 $$Sp\; (a.u.) = - log\left( {\frac{S - D}{{R - D}}} \right)$$

In Eq. (1), *S* is the spectrum recorded when AuNPs are immobilized, *R* is the obtained spectrum when there are no AuNPs onto the optical fiber facet and *D* is the spectrum recorded when the LED is switched off. Sp values were expressed in arbitrary units (a.u.).

### Scanning electron microscopy characterization

To characterize the AuNPs immobilization, a SEM JEOL JSM-6400 (JEOL, Japan) at 10 kV acceleration voltage was used. The facet of the optical fibers were covered with a chromium monolayer of 5 nm by sputtering, for surface metallization. The aggregation ratio was obtained by particle analysis using the public domain software FIJI (ImageJ, National Institutes of Health, USA).

### Fabrication of probes with the same value of A and refractometric measurements

In order to fabricate OF-LSPR probes that presented similar AuNPs compositions, 17 105MMF fibers were functionalized as previously described and then immersed in a stock solution of colloidal AuNPs. Independently of the time of immersion, the immobilization reaction was stopped by removing the fibers from the solution when their Sp maximum values in water reached a certain value in a range from 0.01 to 0.29. Each of these fibers was then immersed in solutions of glycerol in water with consecutively increasing refractive indices (RI): 1.332 (0% glycerol), 1.342 (5% glycerol), 1.349 (10% glycerol), 1.3615 (22.5% glycerol), 1.372 (30% glycerol). Wavelength and maximum values of their respective Sp signals were recorded after immersion for 30 s in each solution, although the changes were immediate after immersion.

## Supplementary Information

Supplementary Figures.

## Data Availability

The datasets generated during and/or analyzed during the current study are available from the corresponding author on reasonable request.
