# Supplementary material for: A method for the controllable fabrication of optical fiber-based localized surface plasmon resonance sensors
Source: Sci Rep. 2022 Jun 10;12:9566. doi: 10.1038/s41598-022-13707-y (PMC9187767; doi:10.1038/s41598-022-13707-y)
Supplement: Supplementary file 1 — Supplementary Figures. [file 41598_2022_13707_MOESM1_ESM.pdf]

SUPPLEMENTARY INFORMATION

A METHOD FOR THE CONTROLLABLE FABRICATION OF OPTICAL FIBER-BASED LOCALIZED SURFACE PLASMON RESONANCE SENSORS

Alba Calatayud-Sanchez[1,3,#], Angel Ortega-Gomez[2,#], Javier Barroso[1], Joseba Zubia[2], Fernando Benito-Lopez[3,4,5], Joel Villatoro[2,6,*], Lourdes Basabe-Desmonts[1,4,5,6,*]

[1] Microfluidics Cluster UPV/EHU, BIOMICs microfluidics Group, Lascaray Research Center, University of the Basque Country UPV/EHU, Vitoria-Gasteiz, Spain.

[2] Department of Communications Engineering, University of the Basque Country UPV/EHU, Bilbao, Spain.

[3] Microfluidics Cluster UPV/EHU, Analytical Microsystems & Materials for Lab-on-a-Chip (AMMa-LOAC) Group Analytical Chemistry Department, University of the Basque Country UPV/EHU, Leioa, Spain.

[4] BIOARABA Health Research Institute, Microfluidics Cluster UPV/EHU, Vitoria-Gasteiz, Spain.

[5] BCMaterials, Basque Center for Materials, Applications and Nanostructures, UPV/EHU Science Park, Leioa, Spain.

[6] IKERBASQUE, Basque Foundation for Science, Bilbao, Spain.

[*]Corresponding authors: lourdes.basabe@ehu.eus and agustinjoel.villatoro@ehu.eus

[#] Authors contributed equally

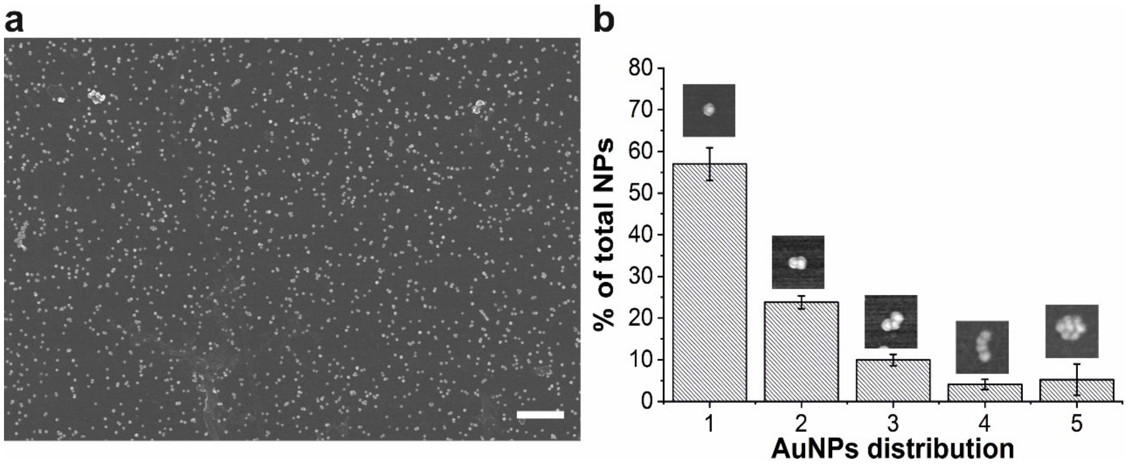

Figure SI 1. Quantification of AuNPs aggregation by SEM image analysis. a) SEM image of an area of 110 μm^2 on the surface of the end face of a functionalized 105MMF after immersion in a AuNPs colloidal solution for 12 min. Scale bar 1 μm. b) Column graph representing the proportion of the total AuNPs present on the fiber facet that were immobilized as a singlet (1), doublet (2), triplet (3), quadruplet (4) or five or more aggregated AuNPs (5), as shown by the insert images. The values are expressed as the mean results from three images of the same fiber, error bars correspond to the standard deviation between those three images (n=3).

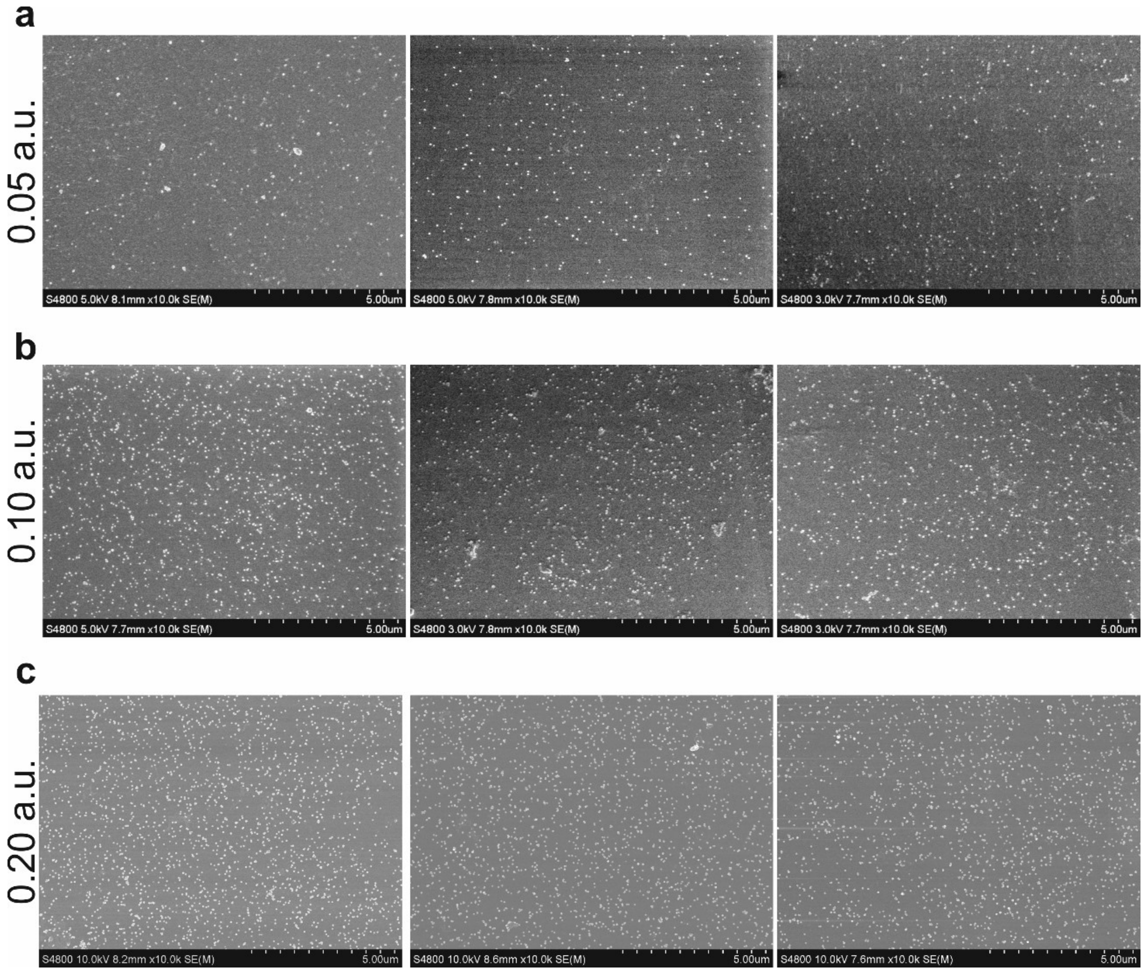

Figure SI 2. SEM image analysis of OF-LSPR with the same Sp maximum values. 110 μm^2 SEM images of the end face surfaces of 9 fibers: three replica of three Sp maximum values: 0.05, 0.10 and 0.20 a.u.

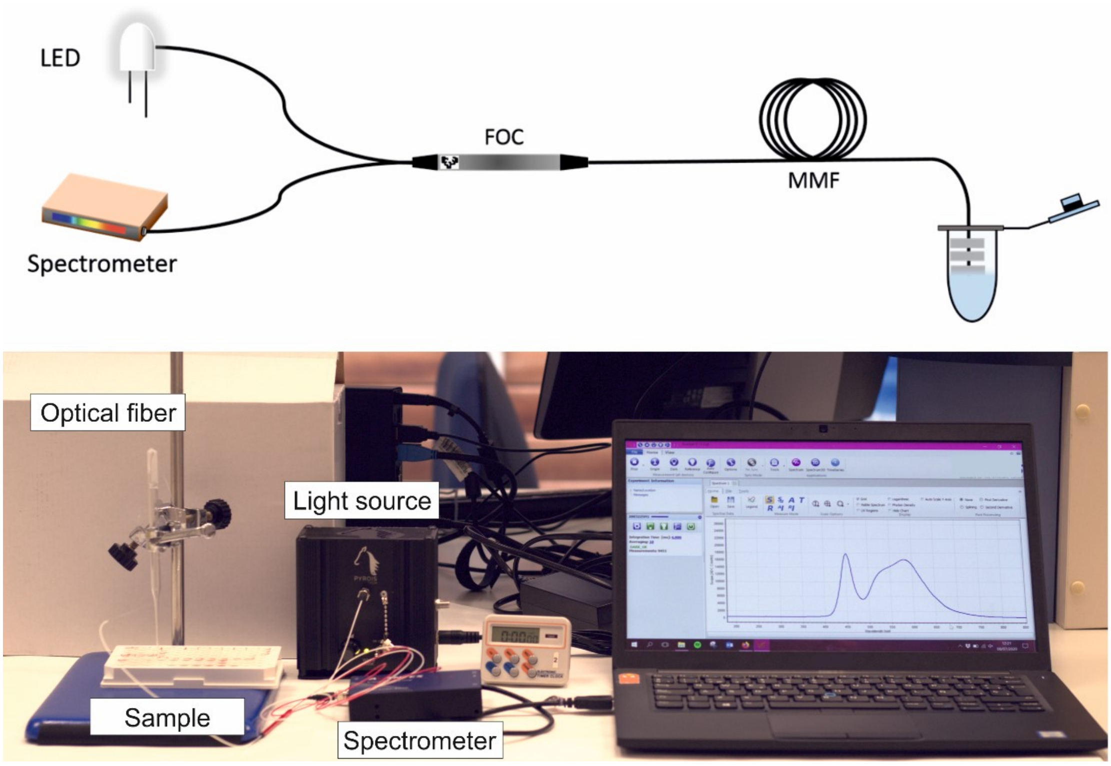

Figure SI 3. Optical set-up. White light is launched from the LED through the FOC to the end face of the optical fiber. The light is reflected by the optical fiber end face, which acts as a low reflectivity mirror. The reflected light passes again through the FOC, finally arriving to the spectrometer and the computer where the evolution of the LSPR signal can be followed in real time.

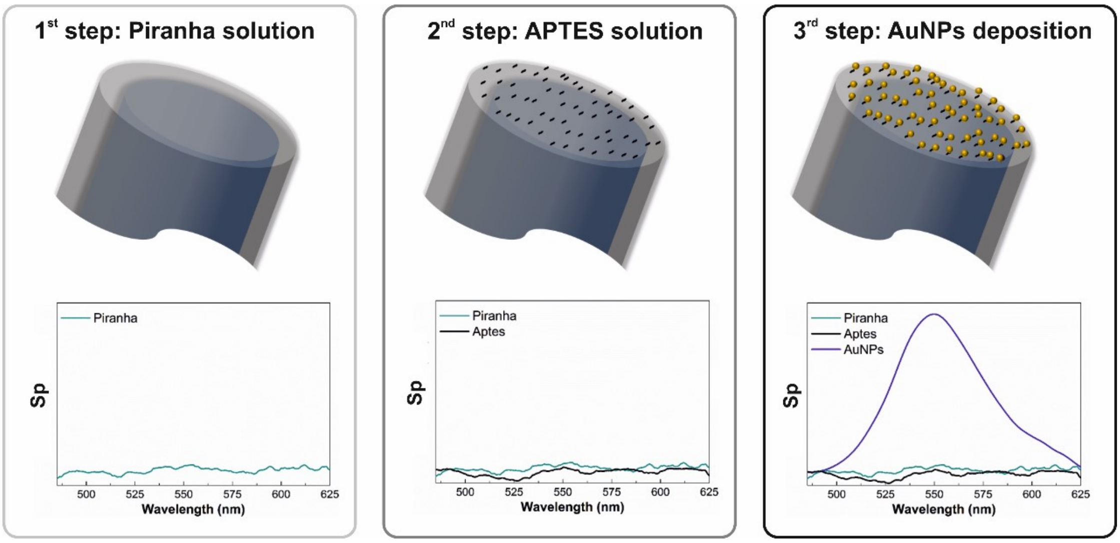

Figure SI 4. AuNPs immobilization process and optical spectrum at each step. The end face of the optical fiber is immersed first in piranha solution for its oxidation and then in a solution of APTES, a molecule that acts as linker for the immobilization of AuNPs to the surface of the fiber as a self-assembled monolayer (SAM). Finally, the fiber is immersed in a suspension of AuNPs. The absorbance spectrum arises from the AuNPs immobilized at the tip of the fibers.
